# Exploring health insurance services in Sudan from the perspectives of insurers

**DOI:** 10.1177/2050312117752298

**Published:** 2018-01-11

**Authors:** Anas Mustafa Ahmed Salim, Fatima Hashim Mahmoud Hamed

**Affiliations:** 1 Faculty of Pharmacy, University of Khartoum, Khartoum, Sudan; 2 Faculty of Economics and Social Studies, University of Khartoum, Khartoum, Sudan

**Keywords:** Social health insurance, private health insurance, benefits packages, quality of services, affordability, sustainability

## Abstract

**Background:**

It has been 20 years since the introduction of health insurance in Sudan. This study was the first one that explored health insurance services in Sudan from the perspectives of the insurers.

**Methods:**

This was a qualitative, exploratory, interview study. The sampling frame was the list of Social Health Insurance and Private Health Insurance institutions in Sudan. Participants were selected from the four Social Health Insurance institutions and from five Private Health Insurance companies. The study was conducted in January and February 2017. In-depth individual interviews were conducted with a convenient sample of key executives from the different health insurers. Ideas and themes were identified and analysed using thematic analysis.

**Results:**

The result showed that universal coverage was not achieved despite long time presence of Social Health Insurance and Private Health Insurance in Sudan. All participants described their services as comprehensive. All participants have good perception of the quality of the services they provide, although none of them investigated customer satisfaction. The main challenges facing Social Health Insurance are achieving universal coverage, ensuring sustainability and recruitment of the informal sector and self-employed population. Consumers’ affordability of the premiums is the main obstacle for Private Health Insurance, while rising healthcare cost due to economic inflation is a challenge facing both Social Health Insurance and Private Health Insurance.

**Conclusion:**

In spite of the presence of Social Health Insurance and Private Health Insurance in Sudan, the country is still far from achieving universal coverage. Moreover, the sustainability of health insurance is questionable. The main reasons include low governmental financial resources and lack of affordability by beneficiaries especially for Private Health Insurance. This necessitates finding solutions to improve them or trying other types of health insurance. The quality of services provided by Social Health Insurance and Private Health Insurance was described as good, but no insurance in Sudan measured customer satisfaction as yet.

## Introduction

Sudan is a lower middle income country. It spends about 6.5% of its Gross Domestic Product (GDP) and 8.2% of the general government expenditure on health. Out-of-pocket share is about 70% (US$84.0 per capita) while the general government heath expenditure represents only 22.3% (US$26.9 per capita).^1^

Prior to 1990s, health services at public healthcare facilities in Sudan were provided mostly free of charge. As a part of major structural economic reforms in 1992, a new financing mechanism based on user fees were introduced.^2^ The impact of the introduction of user fees in public healthcare facilities was not well studied; however, anecdotal evidence suggests that it had significantly affected access and utilization of health services with little or no significant improvement in the availability and the quality of care.^2^ The compensatory mechanisms introduced with the new reforms to act as a safety net for the poor have failed to achieve its goals. Its contribution has been recognized to be very limited as a result of the insufficiency of resources allocated.^2^

It was evident that acquiring loans from relatives and friends is the most widely used method by individuals to address expensive healthcare cost in Sudan. Assistance from relatives represents one of the main sources that enable patients to acquire health service.^2^

Social Health Insurance (SHI) was implemented in Sudan in 1997 as an attempt to overcome the problem of accessibility.^1^ In 2017, SHI reached 71.5% coverage in Khartoum State (966,728 families out of 1,351,514 families) and 50.7% overall coverage in the rest of the states (16,012,805 out of 31,583, 869 individuals). About 5.5% of the population is covered by other health insurance schemes such as police, military and para-statal organizations. Zakat, which is a form of Islamic charity, and Ministry of Finance pay premiums annually for the poor (about 350,000 families), but there is still a huge gap as an estimated 2,300,000 poor families are not covered.^1^

Membership of National Health Insurance Fund (NHIF) is compulsory for the formal sector, while it is voluntary for the informal sector and small companies (≤10 employees). The subscriber unit is the family and beneficiaries include the principal member and dependents (spouse, children and parents). Out of the population covered, 30% are from the formal sector, while the informal sector represents 22.5%, and the remaining belongs to other various sectors. NHIF targets achieving universal coverage by 2031, when over 80% of the population in Sudan is expected to have insurance.^1^

The Federal Ministry of Finance is the major contributor in financing NHIF (72% of funds), followed by the para-statal organizations (12.7%), while households’ contribution is lower (9%). The level of the current households’ contribution was described as far less than it should be, thus impacts the sustainability and the goal of achieving universal coverage. The formal sector contributes 10% of the employees’ salaries, which is deducted at source, 4% from the employee and 6% from the employer. For the informal sector, households pay a flat rate of 40.0 Sudanese Pound (SDG) per household per month.^1^

Khartoum State Health Insurance (HIKS) is geographically limited to Khartoum State. The State Ministry of Health is the major source of financing (36.5%), followed by households (27%). The para-statal organizations pay over 16%, while the private employers’ contribution is about 8.5%. Membership regulations of HIKS are similar to NHIF.^1^

While NHIF provides health services at its own health facilities, the funds collected by HIKS are used to purchase services from outsourced providers. The major providers of services are the primary healthcare facilities of the State Ministry of Health and the Federal Ministry of Health hospitals.^1^

Other insurance schemes in Sudan include police and military insurances. The Ministry of Interior covers members of police service and their dependents and provides services through their own health facilities. Likewise, Ministry of Defense covers the military members and their dependents and provides services through their own facilities^1^ Besides, there are several Private Health Insurance (PHI) companies in Sudan that provide voluntary insurance service, mainly for the employees of private companies and international organizations.

Few published studies investigated health insurance in Sudan. A study conducted in 2009 investigated insurance enrolment and the factors that determine such enrolment. The results showed that among a sample of 72,526 participants, only 14,461 (19.9%) were insured. The enrolment showed regional and socioeconomic disparities. Of the Khartoum participants 34% were found to be insured compared to 14.3% only in Darfur. Moreover, rural–urban disparity was remarkable; 16.9% of the rural population was insured, compared to 25.3% of the urban dwellers. The enrolment was also determined by occupation, as civil service workers had an 80% higher chance of being enrolled compared to those unemployed. People with chronic diseases such as diabetes and hypertension were more likely to be insured than those without such illnesses.^3^

Another study assessed SHI in Khartoum State in 2008. Results showed that 70% of beneficiaries prefer the previous free healthcare system. Although 92% of participants perceived the services provided by SHI as good, 55% of participants were looking for better services even for higher enrolment fees. About 80% of the surveyed population described the referral system as a convenient one, while most of those who described it as inconvenient were from peripheral areas. About 60% of participants described the process of receiving services and medicines dispensing as complicated. The study showed that 70% of the population described the enrolment system as complicated and requires too many documents.^4^ About 11 years after the introduction of SHI in Sudan, Yousif^5^ revealed that the main challenges facing SHI in Sudan are weakness of financing, weak population coverage and difficulties in recruiting the informal sector.

In 2010, a study analysed the potential for PHI to contribute to universal coverage using the Shiekan insurance company as a case study. Results showed that the majority of the insurees are from private enterprises (68%), while public sector insurees represented 32% only. About 74% of respondents described the quality of services as good. The main shortcomings were the lack of coverage of some illnesses and investigations, the high premiums (61% of respondents described Shiekan’s premium as high or very high), the financial ceiling system, and that the list of services providers lacks important doctors. PHI in Sudan also faces the same difficulties of SHI in enrolling informal sector workers.^6^

## The need for the study

Despite the current mix of SHI and PHI, universal coverage has not been achieved in Sudan. We believe that exploring insurers’ insight regarding their perception of the services they provide and the challenges facing them should allow for better understanding of why universal coverage has not been achieved. To the best of our knowledge, this study is the first to explore health insurance in Sudan from the perspective of insurers.

## Methods

This study aimed at exploring health insurance services in Sudan from the perspectives of the insurers. This is a qualitative, cross-sectional exploratory study. The sampling frame was the list of health insurance institutions in Sudan including SHI and PHI (Table 1). A convenience sampling technique was used to select participants. Participants included all SHI institutions in Sudan (NHIF, HIKS, Military and Police insurance) and five PHI companies including Neelain Insurance Company Ltd, Shiekan Insurance and Reinsurance Company Ltd, Islamic Insurance Company Ltd, Blue Nile Insurance Company and United Insurance Company (Sudan) Ltd.

**Table 1.** Health insurance institutions/companies in Sudan.

No	Insurance institute/company	Type
1	National Health Insurance Fund (NHIF)	SHI
2	Health Insurance Corporation of Khartoum State (HIKS)	SHI
3	Military Insurance	SHI
4	Police Insurance	SHI
5	Neelain Insurance Company Ltd	PHI
6	Shiekan Insurance and Reinsurance Company Ltd	PHI
7	Sudanese Company for Insurance and Reinsurance Ltd	PHI
8	Islamic Insurance Company Ltd	PHI
9	United Insurance Company (Sudan) Ltd	PHI
10	Middle East Insurance Ltd	PHI
11	Juba Insurance Company Ltd	PHI
12	Baraka Insurance Company (Sudan) Ltd	PHI
13	Blue Nile Insurance Company	PHI

SHI: Social Health Insurance; PHI: Private Health Insurance.

The study was conducted in January and February 2017. In-depth individual interviews were conducted with a convenient sample of key executives from the different types of health insurance institutions in Sudan. All interviews were conducted by one investigator to improve consistency in data collection. All participants were given information sheet regarding the aim of the study and were asked to sign a written informed consent. Interviews were conducted in the English language. The average length of each interview was between 45 and 60 min.

Participants were asked mostly open questions. A simple checklist of topics (rough topic guide) was used to begin and guide the conversations (Table 2). Probing and subsequent questions were developed during the interviews depending on participants’ answers. Notepapers and audiotape recording were used to collect the data, which was then transcribed by the first author and checked for accuracy by the second author. Transcripts were produced in English and were paper based.^7^

**Table 2.** Interviews rough topic guide questions.

Q1	What is the percentage of population you cover?
Q2	Are there any disparities in coverage among the different socioeconomic or geographical groups?
Q3	Do you provide principal or supplementary coverage? Explain
Q4	What are the benefits packages of your insurance?
Q5	What are the processes for accessing the services?
Q6	What are the excluded services, if any?
Q7	Do you have gatekeeping process for services provision? Explain
Q8	Are there any fees to be paid by the insurees at the points of services delivery?
Q9	Do you have ceilings beyond which services cannot be provided?
Q10	How do you provide the services? At your own facilities or as outsourced?
Q11	How do you perceive the quality of services you provide?
Q12	How do you perceive the convenience of the processes?
Q13	What are the advantages over other schemes in Sudan and other countries?
Q14	What are the areas that need improvement?
Q15	Did you measure customer satisfaction?
Q16	What is the unit of entry?
Q17	What are the premiums to be paid by participants and the enrolment unit?
Q18	How do you perceive the affordability of the premiums?
Q19	What are the main challenges facing you?

The interviews were coded using thematic analysis. Manual coding was used and coding continued till the identification of all themes. The field data were coded by the first and the second author separately. The two coding were then compared and any discrepancies between them were discussed and final codes agreed.^7^ Computerized research records were stored in a password-protected computer, while the paper files data were stored under lock and key. Data were protected effectively against improper disclosure when received, transmitted and stored. This study was approved by the Faculty of Economic and Social Studies, University of Khartoum in January 2017.

## Results

Different themes emerged from the results regarding services provided by health insurance in Sudan, the self-perception of the quality of these services and the challenges facing health insurance. Themes are reported below and supported by verbatim participant quotes identified by interviewee number (e.g. P1, P2, P3 and so on for PHI participants and S1, S2, S3 and so on for SHI participants).

## Pool and equity of coverage

All health insurers in Sudan admitted that their current level of coverage is not satisfactory especially the PHI. HIKS and NHIF reached 71.5% (966,728 out of 1,351,514 families) and 50.7% (16,012,805 out of 31,583, 869 individuals) of population coverage, respectively; however, there are considerable disparities in insurance coverage between rural and urban areas and between formal and informal sectors. Regarding PHI, the current coverage is small and is estimated to be below 500,000 individuals:

Our coverage reached 50.7% only (16,012,805 out of 31,583, 869 individuals). There are disparities between formal and informal sectors, and between rural and urban areas. Recruiting informal sector and ensuring their monthly payment remain as challenges. S3

We currently cover 71.5% of Khartoum State population. S7

Yes, there are disparities regarding the representativeness of different socioeconomic classes in our schemes due to small number of subscribers and high premiums. The total number of insurees is below 500,000 individuals. P26

## Services packages

Concerning services packages, two main themes emerged. For the SHI, there is only one services package for all beneficiaries. On the other hand, PHI companies provide different packages with different premiums. These packages have either different types of services or have the same types of services but with different cost ceilings:

We have different types of packages including bronze, silver, gold, and platinum. They differ from each other in terms of the premiums and the benefits packages. P25

We have four different packages. They do not differ in the benefits, but they differ in the cost ceilings. P31

We have one services package for all participants regardless of their hierarchical positions. S9

## Services coverage

Coverage can be analysed in terms of breadth, depth and height. Breadth indicates coverage in terms of population, depth indicates coverage in terms of services provided and height indicates coverage in terms of the extent of financial protection.^8^

All the study participants described their benefits packages as comprehensive and cover the three levels of healthcare: primary, secondary and tertiary levels. SHI and PHI differ in the process of accessing each healthcare level. In NHIF, HIKS, Military and Police insurances, the patient should consult the General Practitioner (GP) first who has certain list of investigations and medicines to deal with. The GP may then refer the patient to the secondary level. This is called a gatekeeping process. PHI in Sudan allows the patient to access the services at the secondary level from the start.

Both SHI and PHI schemes provide principal coverage. They cover consultations, investigations, operations and hospitals admissions. PHI does not provide supplementary coverage as in developed countries:

We as a SHI offer different types of services for beneficiaries at all healthcare levels including primary, secondary and tertiary levels. The patients should approach firstly the primary level (the GP). The GP may then refer the patient -if necessary- to the secondary level where he can meet the specialist. The tertiary level involves services such as hospitals admissions and operations. This is a gatekeeping process. S1

We provide services at three levels; primary, secondary and tertiary. Each level has a specific list of investigations and medicines. The starting point in our consultation is the GP. This is a WHO standard. There are few exceptions such as the ophthalmic or dentistry healthcare for which the patient can see the consultant from the start. S7

Our services include outpatient services, in-patient services, surgeries, different types of specialties even dental services, ophthalmology, optics, chronic and pre-existing conditions. We have also supplementary insurance services, which are the services that usually not provided by the basic health insurance. P25

We do not have a gatekeeper; the patient is free to see the GP or the consultant. However, certain procedures or services cannot be done or requested by the GP. P27

In general, the package of SHI schemes does not include coverage outside Sudan; however, Military and Police provide some sort of co-payment to their insurees if their conditions necessitate treatment outside the country. Some PHI packages include treatment outside Sudan, and this is usually the high premiums packages:

When the patient needs treatment outside Sudan, and the doctors confirm this, we offer some sort of co-payment. S17

Yes, we cover services in certain countries outside Sudan for certain types of packages. It depends on the cost ceiling of the package. P27

## Services provision

Two themes emerged regarding who provide the services. The NHIF, Military and Police schemes provide the services by themselves at their own healthcare facilities, while HIKS and PHI companies purchase the services from providers:

We provide insurance rather than services, we purchase the services. S3

Our services are outsourced, but we have a system of control. There is a medical services department that monitors services at the points of delivery. S5

We provide the services at our own healthcare facilities. However, if there are some services that are not available, the patient can obtain them from outside our facilities and we provide him with reimbursement. S18

## Contributions and enrolment unit

Enrolment fees vary significantly between governmental SHI and PHI. The latter is much more expensive compared to SHI.

In SHI, the enrolment unit is the households and the contribution fees are 10% of employee salary per month for the formal sector (6% to be paid by the employer and 4% by the employee). For the informal sector, the fees per household is 40 SDG per month regardless the number of family members. For PHI, the fees depend on the package chosen and have a wide range. The industry average ranges between 1500 and 6000 SDG per person per annum. The enrolment unit in PHI is the individual; moreover, they enrol only institutions and enterprises:

The enrolment fees for the formal sector are 10% of the employee salary, 6% to be paid by the employer and 4% by the employee. For the informal sector it is 40 SDG per household per month regardless of the household number. S3

The premiums depend on the purchased package. All services and procedures are provided on credit basis. The fees we charge compared to the services we provide is reasonable. The industry average is between 1500–6000 SDG per person annually. P23

We do not enroll individuals or families; we enroll institutions or enterprises only. P29

For all health insurance in Sudan, patients receive the services for free at health facilities except the medicines, for which the patient pays a percentage of the cost depending on the insurance type and package. In SHI, the patient pays 25% of the medicines cost while in PHI this depends on the package. In general, it is between 0% and 10%:

In our scheme, the patient has to pay 25% of the medicines cost. S12

This varies, in the best packages, the patient does not pay for medicines, while in other lower premiums packages, the patient pays 10% of medicines cost with an annual ceiling. P28

## Medicines prescribing and dispensing

Two themes identified regarding how insurers deal with medicines brand names at the level of dispensing. SHI considers only generic names with the exception of medicines that have narrow therapeutic indexes and their pharmacokinetic profiles vary between brands, while PHI considers brand names upon dispensing:

We dispense medicines according to generic names only except for the medicines that have narrow therapeutic indexes and their blood levels are affected by switching from a brand to another such as digoxin. S9

We dispense prescriptions according to the prescribed brand names. P30

In almost all types of health insurance in Sudan, GPs are not allowed to prescribe all medicines. The excluded medicines are either those highly specialized or those with high cost:

GPs are allowed to deal with specified lists of medicines and investigations, their lists are more limited compared to consultants’ lists. S6

Some medicines can be prescribed by GPs while others should be prescribed by consultants only or even at specialized healthcare institutions. P29

## Excluded services

Different themes were identified regarding the services that excluded from insurance coverage. NHIF and HIKS exclude certain expensive services such as open heart surgery, cancer therapy and renal transplant. Participants explained that other governmental bodies provide co-payment for such types of patients. The two other governmental health insurances (Military and Police schemes) do not exclude these services.

Military and police insurances cover all registered medicines in Sudan, while PHI, HIKS and NHIF have lists of medicines that to be covered:

There are certain expensive services that we do not cover such as renal transplant, open heart surgery and knee replacement surgeries. However, we provide partial support for those patients. S5

We do not exclude expensive services such as open heart surgery; actually we have a contract with the heart center to manage our patients. S16

There are complete sets of medical diagnosis and treatments that are excluded. P30

We have no list of excluded medicines except mental care and supplements. Excluded services include nursing at home, HIV treatment, infertility treatment and non-registered medicines. P27

## Services’ financial ceilings

SHI and PHI differ regarding services’ financial ceilings. While SHI schemes have no financial ceiling for services provision whatever the costs, PHI has total financial ceiling for each package and ceilings for operations, medications, hospitals admissions and others:

We have ceilings for patients. The ceiling depends on the contract. We have a total services ceiling and also ceiling for each type of services such as a ceiling for chronic illnesses, a ceiling for opticals, and a ceiling for operations, etc. P26

We do not have financial ceilings for our patients. The patient enjoys healthcare services that might be much higher than his contribution. S2

## Perceptions of the quality of services

One theme emerged regarding perceptions of the quality of services provided. All participants showed good perception of the quality of services they provide. However, none of them conducted customer satisfaction surveys:

There is a study that was conducted by University of Khartoum in different states. The level of customers’ satisfaction emerged from the study was more than 80% as far as I remember. S1

I think we provide good quality services. For the primary and secondary levels, we are almost equivalent to PHI, but we lag behind them in the tertiary level as the privately insured individuals can be admitted at high quality private hospitals. S4

Compared to SHI in other countries in the region, Egypt for example, we are much better in terms of services quality. S11

I think we provide good quality services. We have different advantages over governmental insurance including admission to private hospitals, flexibility in consulting specialists from the start and coverage outside Sudan. We have a convenient automated system regarding approval letters. P29

We did not measure customer satisfaction as yet; however, I think there is a good level of satisfaction. P28

We are currently in the process of developing quality indicators in order to assess the quality of services objectively. S5

## Convenience of the processes

All insurers described that their processes are convenient. They have large networks of health centres and everything can be done easily at the healthcare facilities with few exceptions that need approval letters such as operations and expensive radiology investigations. In HIKS, approval letters should be taken from counties; this is inconvenient for the patients. However, HIKS started to implement an automated system in few healthcare facilities to ease the process of approval letters. PHI used to have an automated system for approvals:

In case of operations the patients do not need to come to the headquarter, as there is health insurance administration at each county. Some services need approvals such as MRI, CT scan, and the patient can take approval letter from his county. The hospital admission process in case of emergencies doesn’t need an approval letter. We are now working on an automated system; the approvals will be done through the system, so the patient does not need to go to his county. S5

Our referral system is convenient. The different levels of services can be found at each center. However, we applied the WHO standard that the GP cannot refer more than 10% of the cases. S6

Our services are convenient. We have a large network of coverage that includes all states of Sudan. Moreover, if the patient receives services outside our network, we reimburse him with 80% of what he paid. P25

## Challenges

Participants described several challenges that have been facing health insurance in Sudan. For the governmental SHI, the main challenge is the financing as the contribution paid by the beneficiaries is low and most of the finance is from the Ministry of Finance or the Ministry of Health, which may affect the sustainability.

Other challenges that face all types of health insurance in Sudan include economic inflation which escalates the cost of the services leading to financial losses to insurers, recruitment of the self-employed and informal sector, and the problem of medicines stock-outs in the country in recent years due to lack of hard currency needed for medicines importation:

There are different challenges including how to covers self-employed and non-formal sector, how to ensure the sustainability of their payments and how to access them. Challenges also include availability of medicines and the inflation which affected our resources. The enrolment contribution has not been changed in the past five years while there is a considerable increase in the cost due to inflation. S1

The main challenge that we are facing is the financing. Some experts predicted that we will collapse by 2019 due to financial issues. S7

The insurance market is small in Sudan. The culture of insurance yet to be rooted in the mindset of the Sudanese. The cost of medical services constitutes the main challenge. P12

Economic instability (inflation) is the main problem. Usually insurance companies predict 10–20% increase in medicines prices annually and accordingly determine their premiums. In the previous two years, the increase in medicines prices was substancial which affected our expenses significantly leading to losses. P30

## Discussion

This study showed the current mix of SHI and PHI in Sudan did not reach universal coverage after 20 years of implementation. Moreover, there are disparities regarding insurance coverage between rural and urban areas and between formal and informal sectors. It is questionable that NHIF in Sudan is able to reach universal coverage with its current combination of comprehensive coverage and limited financial resources. To solve such an issue without affecting the comprehensiveness of the services provided, the premiums for the household need to be increased significantly which is a challenge in a country that 46.5% of its population is below the poverty line.^9^

The relatively better enrolment achieved by HIKS in Khartoum State (71.5% coverage) can be attributed to lower governmental contribution (36.5%) and higher contribution of households (27%) in its financing compared to NHIF. Besides, it is easier to reach and recruit people in a single state compared to large number of states with high proportion of rural areas that covered by NHIF.

To address the issue of the low enrolment of informal sector, the government should develop mechanisms to recruit informal workers. Besides, it should ease the process of enrolment and reach them at their place of work. Also it may take bold step such as making health insurance a condition for attending public healthcare facilities with the exception of emergencies. To convince those who live in rural areas, especially remote ones, the government should play an active role in reaching them and conducting awareness campaigns using different channels including TV, radio and social media. Moreover, improving the quality of the services, the convenience of processes and the quality of the healthcare facilities will encourage people to seek health insurance.

Despite the high percentage of out-of-pocket spending in Sudan and inadequate coverage by SHI, the current number of population insured by PHI is small; this is might be due to high premiums as described in a previous study.^9^ Other reasons may include Sudanese culture, as people used to help each other in case of catastrophic health expenditure; besides, lack of awareness of the benefits of the insurance or mistrust in insurance providers might have played a role.

Other schemes can be tried in Sudan to address the gaps of SHI and PHI such as the mutual and community health insurance (CHI). This type of insurance is considered the most common form of voluntary insurance in developing countries. It is a set of households with roughly similar prior health risk who forms a cooperative or mutual insurance arrangement. The bulk of the revenue for such an organization would come from its customers as premiums. With a combination of trust and careful, enforceable contract language and proper management, this type of insurance can make a success. However, the reasons that led to inadequate coverage by CHI in many developing countries should be studied carefully.

Another possible device for furnishing insurance is a not-for-profit firm that charges premiums, absorbs any excess losses and retains any surplus of premiums over benefits. The control or management of the nonprofit firm can come from a variety of sources, as can the internally generated revenues to furnish equity capital.^10^

Universal coverage is rarely to be found in developing countries with few exceptions such as SHI in China, which successfully achieved universal coverage in 2011, representing the largest expansion of insurance coverage in human history. There are major drivers for China’s success, including strong public support for government interventions in healthcare, political commitment from top leaders, heavy government subsidies, fiscal capacity backed by China’s economic power, financial and political responsibilities delegated to local governments and programmatic implementation strategy.^8^

Findings of inadequate coverage especially for the informal sector were reported in many developing countries.^11^ The WHO review that included 82 non-profit CHI of the informal sector in developing countries found that very few of these schemes cover large population. The median value of the percentage of the eligible population covered was found to be 24.9%. Besides, many people tended to sign up with the CHI at the moment of illness; thus, the members with high risks were over-represented leading to adverse selection.^11^ Low percentages of enrolment were also observed in a study on 5 CHI in East and Southern Africa. Enrolment percentages were between 0.3% and 6.5% of the target population.^12^ Another study that was conducted in nine West and Central African countries (WCA Study) on 22 CHI showed that most of the schemes have low enrolment with percentages between 8% and 53%.^13^

The WHO study concluded that very few schemes reached the vulnerable population groups, unless government or others facilitated their membership through subsidies.^14^ In the Bwamanda scheme in Zaire, participation across income groups was not equal. The very poor and the high income groups were less well represented in the member population as compared to the non-member population.^15^ In the Rwanda project, however, there was no statistically significant difference regarding the enrolment across different income groups.^16^

Our study showed that all participants from the different schemes have good perception of the quality of the services they provide. A previous study in Khartoum State assessed the services provided by HIKS and showed that 92% of participants were satisfied with the quality.^4^

There are two surveys conducted by Sudanese newspapers that assessed the quality of the services provided by NHIF and HIKS from the perspectives of the insurees. The results identified many aspects of customers’ dissatisfaction. In these surveys, patients described different problems including unavailability of specialists at NHIF centres, exclusion of high cost medicines from the insurance list, long distance to the services provision centres, inconvenient processes and the problem of referral from other states to Khartoum which highlighted a lack of integration between NHIF and HIKS.^17,18^ It is important for the insurers in Sudan to measure customer satisfaction from customers’ perspectives in order to identify the gaps in services provision.

The quality of care was highlighted in similar schemes outside Sudan. For example, in the Maliando scheme in Guinea-Conkary, lack of quality of care was described as the most important cause of non-enrolment.^19^ Attitudes towards the CHI scheme were also assessed in Hanang District, Tanzania. One of the reasons for non-enrolment was that members did not have access to good quality of care at CHI health facilities.^20^

This study showed that all insurers in Sudan offer principal coverage and that their services constitute comprehensive packages. They differ in terms of the presence or absence of gatekeeping process.

SHI in Sudan has essential generic drug policies, while this is not the case in PHI. This model of providing principal coverage by the two types of insurers is useful for the time being in Sudan due to low coverage of SHI from one hand and the concerns regarding the quality of healthcare facilities of SHI from the other hand. Upon achieving universal coverage by SHI and improving the public healthcare facilities, PHI may switch to the provision of the supplementary coverage. Measures such as gatekeeping process and dispensing medicines by generic name implemented by SHI in Sudan should help in reducing the cost of medical care and prevent providers’ moral hazards.

The WHO study stated that the overall benefits packages were only weakly defined.^14^ Although some schemes defined exclusions, there was a tendency to include all available services at facilities. With this broad approach, enrolment rates among patients with pre-existing conditions, especially chronic illnesses, tended to be high. Gatekeeping practices were found to be the case in Bwamanda Health Insurance Scheme, whereby patients could only get access to (insured) hospital care after being referred by a primary healthcare centre.^15^

Information on 67 mutual health organizations in the WCA Study showed that only 4 schemes had introduced essential and generic medicines policies and only 2 of the 15 schemes whose benefits packages contained both primary and in-patient care had introduced mandatory referral for benefits beyond the primary care level.^13^ In the Rwandan project, services covered include preventive and basic curative care by nurses, essential medicines, hospitalization at the health centre and ambulance transfer to the district hospital.^16^ In China, the New Rural Cooperative Medical Scheme (NCMS) covers both outpatient and in-patient care in about 70% of the counties. The other 30% offer coverage for in-patient care only.^8^

This study showed that SHI has a flat-rate contribution, which is constant for all families regardless of its economic status. Flat-rate contribution may impact poor families and acts as a barrier for their enrolment. From the perspectives of SHI participants, contribution fees were described as affordable. The level of the current contribution was described as far less than it should be according to the actuarial study conducted in 2013, which is expected to impact the sustainability of the insurance and the goal of achieving universal coverage.^1^

Regarding PHI, the Shiekan study showed that only people with high income or those insured by their employers can join PHI.^6^ PHI in developing countries is usually restricted to population from high income socioeconomic group. In South Africa, for example, 80% of all people with PHI are estimated to belong to the two highest income quintiles while only 2% of the lowest income quintile has PHI.^21^

A number of schemes in the WHO study had analysed the issue of affordability. For the Nkoranza scheme in Ghana, the estimated cost of contributions varied from 5% to 10% of annual household budgets. It was recognized that such contributions could be a financial obstacle to membership. Contributions are also generally levied as flat sums, which is a disadvantage for the poorest: flat contributions are regressive, a flat-rate contribution as a percentage of income being higher for poor than for the non-poor.^14^ In the Rwandan project, membership varied from 5.6% to 7.7% in the lowest and highest income category, respectively; yet, this difference was found not to be statistically significant. One indication, though in this study that affordability matters, is that large households with more than five members had a greater probability to enrol than others.^16^ The explanation given is that contributions were kept flat, irrespective of household size up to seven members; the average contribution per household member was therefore less than for smaller families, inducing greater enrolment. The Thiès Study in Senegal showed that the self-reported poor households had a lower probability to join a CHI than the higher income households.^22^

In SHI in Sudan, Zakat and Ministry of Finance pay premiums annually for the poor (about 350,000 families).^1^ Only a minority (13) of the 44 schemes surveyed in the WHO study had exemption policies to allow the poor households to join.^14^ In one of the three districts in the Rwandan project, the local church paid for the contributions of about 3000 orphans and widows with their family members.^23^

One scheme that from the start introduced a pro-poor policy is the Gonoshasthaya Kendra in Bangladesh. The scheme differentiated contributions according to one of four socioeconomic groups (the destitute, poor, middle-class and rich). An important finding is that the membership rates among the two lowest socioeconomic groups are substantially higher than in the other groups.^24^

All schemes in Sudan consider the family as the unit for enrolment to avoid adverse selection. Achieving adequate membership rates is likely to be easier when households are taken as the basis of membership. In the WHO Study, almost half of the schemes surveyed had the family as the unit of membership.^14^ A number of schemes had actually switched to this type of membership after experiencing problems of adverse selection, as a result of families signing up ill family members or family members most prone to consume healthcare. Also, most of the case studies (14) reviewed in the WCA study had an automatic family coverage.^13^

Our study highlighted the issue of sustainability of health insurance in Sudan. For the SHI, financing is the main challenge for sustainability as the Ministry of Finance is the main contributor in an economy of limited resources and the rising prices of health services due to inflation. Moreover, the comprehensive package increases the cost of the services.

In the WHO Study, a number of reasons for poor financial viability were identified, including the small scale of a CHI, the occurrence of adverse selection and important administrative costs. The WHO Study proposes a useful indicator of overall sustainability, namely the lifespan of a CHI. Of the 37 scheme studied, 27 schemes had an average lifespan of 8.5 years.^14^

## Research limitations

This research did not measure the services provided by health insurance in Sudan from insurees’ perspectives, nor from services providers’ perspectives such as doctors, pharmacists and other healthcare professionals. This is a potential area for future researches. This study explored the challenges facing health insurance from insurers’ perspectives. It is necessary to explore insurees’ perspectives too in this regard as there might be other reasons that lead to low enrolment such as consumers distrust in insurers, misperceptions of the risks and costs of illness, or satisfaction with the social system in Sudan in which relatives, friends and neighbours provide support in case of catastrophic health budget. We believe that conducting in-depth interviews – rather than questionnaires used in previous studies – with insured and non-insured people would add to the knowledge regarding consumers’ perspectives and will assist SHI and PHI in shaping their services accordingly.

## Conclusion

This study aimed at exploring the services provided by the different health insurance schemes in Sudan from the perspectives of insurers. It was found that in spite of the availability of the two insurance types, the country is still far from achieving universal coverage due to financial constraints of the government and lack of affordability by beneficiaries. Moreover, there are disparities in coverage between formal and informal sector, urban and rural areas and between different states.

The benefits packages provided by SHI and PHI are comprehensive, covering primary, secondary and tertiary levels. Such comprehensive coverage might affect the sustainability of the project and the achievement of universal coverage since most of the finance is provided by Ministry of Finance or Ministry of Health in a country with limited financial resources.

The SHI system has a well-defined gatekeeping referral system and medicines policy which might reduce moral hazards. But this system is inconvenient regarding obtaining approval letters from the counties for several types of services. Automated electronic system should be implemented in order to avoid this.

The study showed that insurers have good perception of the quality of services they provide. However, no insurance conducted surveys to assess the quality of services from customers’ perspectives.

Resembling PHI in developing countries, PHI in Sudan provides principal coverage rather than supplementary coverage that found in developed countries. It provides better services compared to SHI especially in terms of the better quality of the private healthcare facilities, automated approval letters and the absence of gatekeeping process. Moreover, some packages involve treatment coverage outside Sudan. However, PHI is much costly compared to SHI and has ceilings for each type of service. Moreover, its main consumers are limited to those with high income or those insured by their private employers.
